# Extracellular Membrane Vesicles and Phytopathogenicity of *Acholeplasma laidlawii* PG8

**DOI:** 10.1100/2012/315474

**Published:** 2012-11-28

**Authors:** Vladislav M. Chernov, Olga A. Chernova, Alexey A. Mouzykantov, Natalija B. Baranova, Oleg V. Gorshkov, Maxim V. Trushin, Tatiana N. Nesterova, Anastasia A. Ponomareva

**Affiliations:** ^1^Kazan Institute of Biochemistry and Biophysics, Russian Academy of Sciences, P.O. Box 30, Kazan 420111, Russia; ^2^Department of Genetics, Kazan Federal University, Kremlyovskaya 18, Kazan 420008, Russia

## Abstract

For the first time, the phytopathogenicity of extracellular vesicles of *Acholeplasma laidlawii* PG8 (a ubiquitous mycoplasma that is one of the five common species of cell culture contaminants and is a causative agent for phytomycoplasmoses) in *Oryza sativa* L. plants was studied. Data on the ability of extracellular vesicles of *Acholeplasma laidlawii* PG8 to penetrate from the nutrient medium into overground parts of *Oryza sativa* L. through the root system and to cause alterations in ultrastructural organization of the plants were presented. As a result of the analysis of ultrathin leaf sections of plants grown in medium with *A. laidlawii* PG8 vesicles, we detected significant changes in tissue ultrastructure characteristic to oxidative stress in plants as well as their cultivation along with bacterial cells. The presence of nucleotide sequences of some mycoplasma genes within extracellular vesicles of *Acholeplasma laidlawii* PG8 allowed a possibility to use PCR (with the following sequencing) to perform differential detection of cells and bacterial vesicles in samples under study. The obtained data may suggest the ability of extracellular vesicles of the mycoplasma to display in plants the features of infection from the viewpoint of virulence criteria—invasivity, infectivity—and toxigenicity—and to favor to bacterial phytopathogenicity.

## 1. Introduction

*Acholeplasma laidlawii *is a unique mycoplasma species (class Mollicutes) from the viewpoint of its adaptation abilities. This bacterium is widely distributed in nature and is one of the five common species of cell culture contaminants and is a causative agent for phytomycoplasmoses [1–3]. Control of mycoplasma infections presents a serious problem whose resolution is connected with clarification of mechanisms of mycoplasma adaptation to the environment, formation of the “parasite-host” system, and realization of virulence. [2, 4]. Sequencing of *A. laidlawii *genome [5] provided an opportunity for application of postgenomic technologies for detection of molecular-genetic fundamentals of mycoplasma survival in different environments [2]. As a result of proteomic-transcriptomic analysis and nanoscopy studies, stress-reactive proteins and genes of *A. laidlawii *PG8 were identified, and it was presented that adaptation of the mycoplasma to unfavorable condition was connected with secretion of extracellular membrane vesicles (EMVs) [2]. It was found that EMVs of *A. laidlawii*, apart from a membrane, contain nucleotide sequences of some genes and display a high mutagenicity toward cells of higher eukaryotes [6]. It is known that EMVs mediate secretion in bacteria, participate in signaling, intercellular interactions, and pathogenesis [7]. It was suggested that EMVs may make a significant input into phytopathogenicity of bacteria [8–10] including mycoplasmas [2]. However, data on phytopathogenicity of bacterial EMVs are absent in the literature. Therefore, investigation of EMVs phytopathogenicity from the viewpoint of virulence criteria—invasivity, infectivity and toxigenicity—toward plants of *Oryza sativa* L. was the aim of the present study.

## 2. Materials and Methods

*A. laidlawii* PG8 strain obtained from the N.F. Gamaleya Institute of Epidemiology and Microbiology (Moscow) was used in this work. The mycoplasma cells were grown in a liquid modified Edward's medium (tryptose, 2% [w/v]; NaCl, 0.5% [w/v]; KCl, 0.13% [w/v]; Tris base, 0.3% [w/v]; horse serum, 10% [w/v]; yeast extract, 5% [w/v]; glucose solution, 1% [w/v]; benzylpenicillin [500,000 IE/mL], 0.2% [w/v]).

Isolation of membrane vesicles from *A. laidlawii* PG8 culture was performed according to Kolling and Matthews [11], with some modifications taking into account features of cell biology and cultivation of mycoplasmas [4].

Rice seeds (*O. sativa* L. breed “Lougovoy”) were sterilized with 0.01% solution of KMnO_4_ for 30 min and then washed extensively with distilled water. The plants were grown under sterile conditions [12] at 27°C (12 h : 12 h light : dark photoperiod and a light intensity 6 klux). Rice plants were infected with *A. laidlawii* PG8 cells and EMVs under sterile conditions as described by Chernov et al. [2] using a spontaneous infection of 10-day plant seedlings through the root system. Plant roots were incubated continuously in Murashige and Skoog medium containing cells or EMVs of *A. laidlawii* PG8. Control plants were incubated in the mycoplasma-free medium. Analysis of the samples was performed since 2 h to 9 days later.

Transmission electron microscopy (TEM) was done with a JEM-1200EX microscope (Japan) according to Chernov et al. [2].

To prepare samples for atomic force microscopy (AFM) studies, EMVs of* A. laidlawii* PG8 were placed onto the mica (Advanced Technologies Center, Moscow, Russia) with the upper layer removed. EMVs were air dried and then rinsed twice with redistilled water, and after each rinsing, the samples were air dried in both instances. AFM imaging was performed with a Solver P47H atomic force microscope (NT-MDT, Moscow, Russia) operating in the tapping mode using fpN11S cantilevers (*r* ≤ 10 nm, Advanced Technologies Center, Moscow, Russia). The height, Mag (signal from lock-in amplifier), RMS (signal from RMS detector), and phase (signal from the phase detector) were performed with the Nova 1.0.26 RC1 software (NT-MDT). The scan rate was 1 Hz. Image resolution was 512 × 512.

DNAs from mycoplasma cells and plant tissues were isolated according to [13]. DNA from EMVs was isolated using commercial kit “DNA-express” (“Litekh”, Moscow). Before the extraction of nucleic acids, samples of EMVs of the mycoplasma were treated with DNAse I (at 37°C for 30 min).

PCR primers were constructed in NSF “Litekh” (Moscow, Russia) using the nucleotide sequences of A. laidlawii PG8-A genes (GenBank accession number NC_010163): ftsZ (Ala1F 5′-ggtttttggatttaacgatg-3′ Ala1R 5′-gcttccgcctcttttattt-3′), pdhC (Ala9F 5′-aaagcaagaccataaggagg-3′ Ala9R 5′-tggagcctgtgtttgttga-3′), pnp (Aq1F 5′-aagcccattgcgatacctgc-3′ Aq1R 5′-ggtgctttaggagaacgtgct-3′), tufB (Aq3F 5′-ccaggtcacgctgactatgtt-3′ Aq3R 5′-acgagtttgtggcattggac-3′), rpoB (Aq6F 5′-tggcatatcttctcttggtaaa-3′ Aq6R 5′-tggcatatcttctcttggtaaa-3′), spacer 16S–23S of ribosome operon (A16LF 5′-ggaggaaggtggggatgacgtcaa-3′ A23LR 5′-ccttaggagatggtcctcctatcttcaaac-3′).

PCR was performed in the following regime: for primers Ala1, 95°C, 3 min (95°C, 20 sec; 52°C, 20 sec; 72°C, 20 sec) (30 cycles); 72°C, 10 min. For primers Ala9, 95°C, 3 min (95°C, 15 sec; 55°C, 10 sec; 72°C, 10 sec) (30 cycles); 72°C, 10 min. For primers Aq1, Aq3, Aq6, 95°C, 3 min (95°C, 5 sec; 63°C, 5 sec; 72°C, 5 sec) (35 cycles); 72°C, 5 min. For primers A23LR, 95°C, 3 min (95°C, 5 sec; 63°C, 5 sec; 72°C, 20 sec) (30 cycles); 72°C, 5 min.

Sequencing of DNA was performed using BigDyeTerminator v3.1 Cycle Sequencing Kits (“Applied Biosystems,” USA) and DNA-analyser 3130 Genetic Analyser (“Applied Biosystems,” USA). Analysis of the nucleotide sequences was done in Sequencing Analysis 5.3.1 software (“Applied Biosystems,” USA) and NCBI (National Center for Biotechnology Information, http://blast.ncbi.nlm.nih.gov/Blast.cgi) database.

## 3. Results and Discussion

The presence of nucleotide sequences of some mycoplasma genes within EMVs of *A. laidlawii *PG8 allowed a possibility to use PCR to perform differential detection of the corresponding structures of the bacterium in samples under study. Despite growth conditions, we detected sequences of genes for *pdh*C, *rpo*B, *pnp,* and *tuf*B genes in EMVs, but not for *fts*Z and 16S–23S rRNA genes. This allowed us to use a combination of primers for differential detection of cells and membrane vesicles in the tested samples.

**Figure 1:**
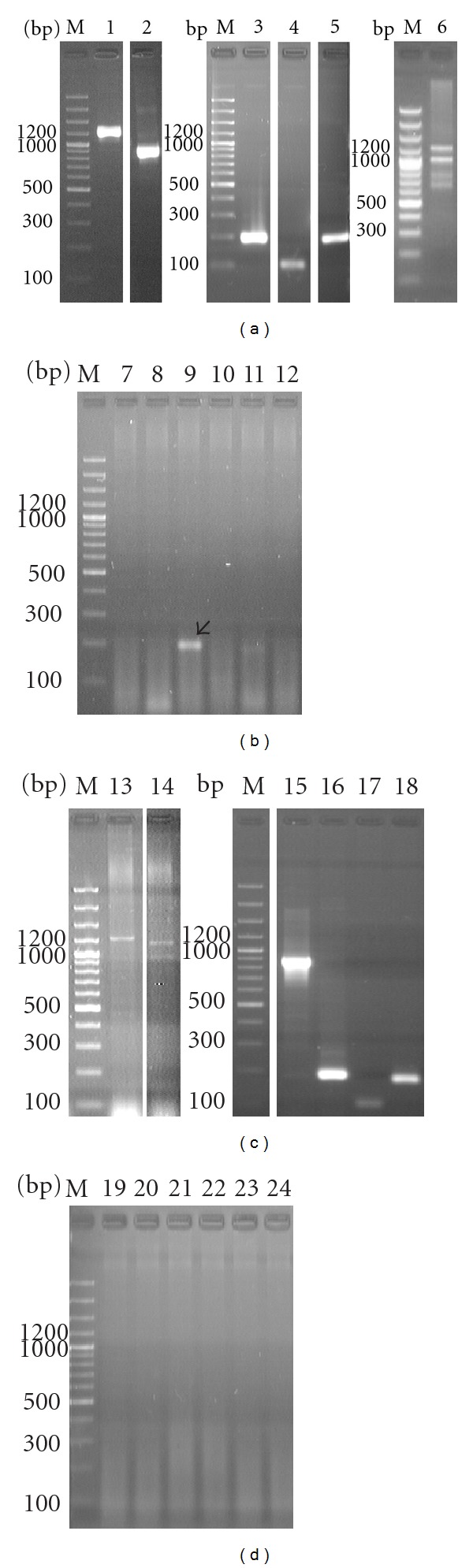
Electrophoregrams of amplification products for DNA nucleotide sequences of the following genes* fts*Z: (1, 7, 13, 19), *pdh*C (2, 8, 15, 20), *pnp *(3, 9, 16, 21), *tuf*B (4, 10, 17, 22),* rpo*B (5, 11, 18, 23), and 16S–23S rRNA (6, 12, 14, 24) *A. laidlawii *PG8 obtained at use, in PCR, total DNA (as matrix) isolated from cells of *A. laidlawii *PG8 (a) and plant leaves grown in medium with the mycoplasma cells (b and c—2 h and 9 days, resp.) and in the absence of the infect (d). Arrow indicates location of the corresponding PCR-signal. M—a marker of fragment lengths.

**Figure 2:**
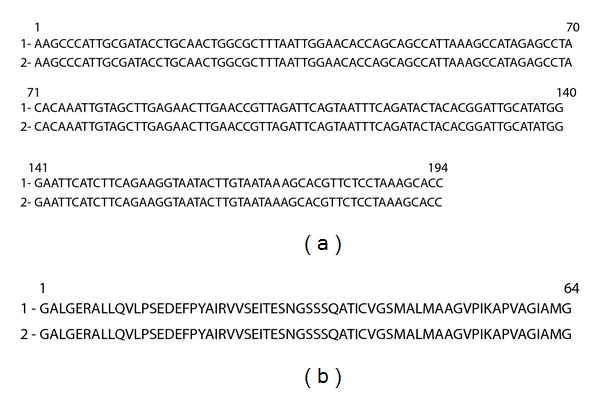
Results of alignment of the nucleotide (a) and amino acid (b) sequences of the fragment of *pnp*-gene of *A. laidlawii* PG8 (1) as well as amplicon (2) obtained as a result of PCR with primers for detection of the nucleotide sequence of *pnp*-gene of *A. laidlawii* PG8 when DNA of plants grown in medium with the mycoplasma cells was used as a matrix. Sequences of forward and reverse primers are indicated with italics.

Previously [2], we reported about the ability of *A. laidlawii* PG8 cells to enter through the root system of *O. sativa* L. into overground parts of plants and induce there changes in ultrastructural organization. This conclusion was based on data of TEM and PCR obtained 9 days after rice cultivation with mycoplasma cells. As a result of use of primers for amplification of the nucleotide sequences for the following genes—*pdh*C (codes dihydrolipoamide acetyltransferase), *rpo*B (codes *β*-subunit of RNA-polymerase), *pnp *(codes polyribonucleotide-nucleotidyl transferase, a global regulator of virulence in phytopathogenic bacteria [14]), and *tuf*B (codes elongation factor Tu) permitting to detect cells as well vesicles of the mycoplasma, and primers for amplification of the nucleotide sequences of *fts*Z gene (codes FtsZ, a protein for cell division), and 16S–23S rRNA gene (codes a spacer zone and flankings) permitting to detect cells but not bacterial vesicles, we found that PCR signals were presented for all indicated genes in tissues of plant leaves grown in medium with mycoplasma cells for 1–9 days (Figure 1(c)). Also, amplicon specific to *pnp *gene of the mycoplasma was found 2 hours since the beginning of plant incubation with mycoplasma cells (Figure 1(b)). Sequencing results confirmed the belonging of a sequence of this amplicon to *A. laidlawii* PG8 *pnp*-gene (Figure 2). The obtained data allow to suggest that (1) EMVs are able to display virulent features linked with infectivity and invasivity in plants; (2) penetration of EMVs to plant tissues precedes to plant infection with mycoplasma cells; (3) EMVs are heterogeneous on the content and functions; (4) virulent features connected with infectivity and invasivity are different in vesicles varying on the content.

**Figure 3:**
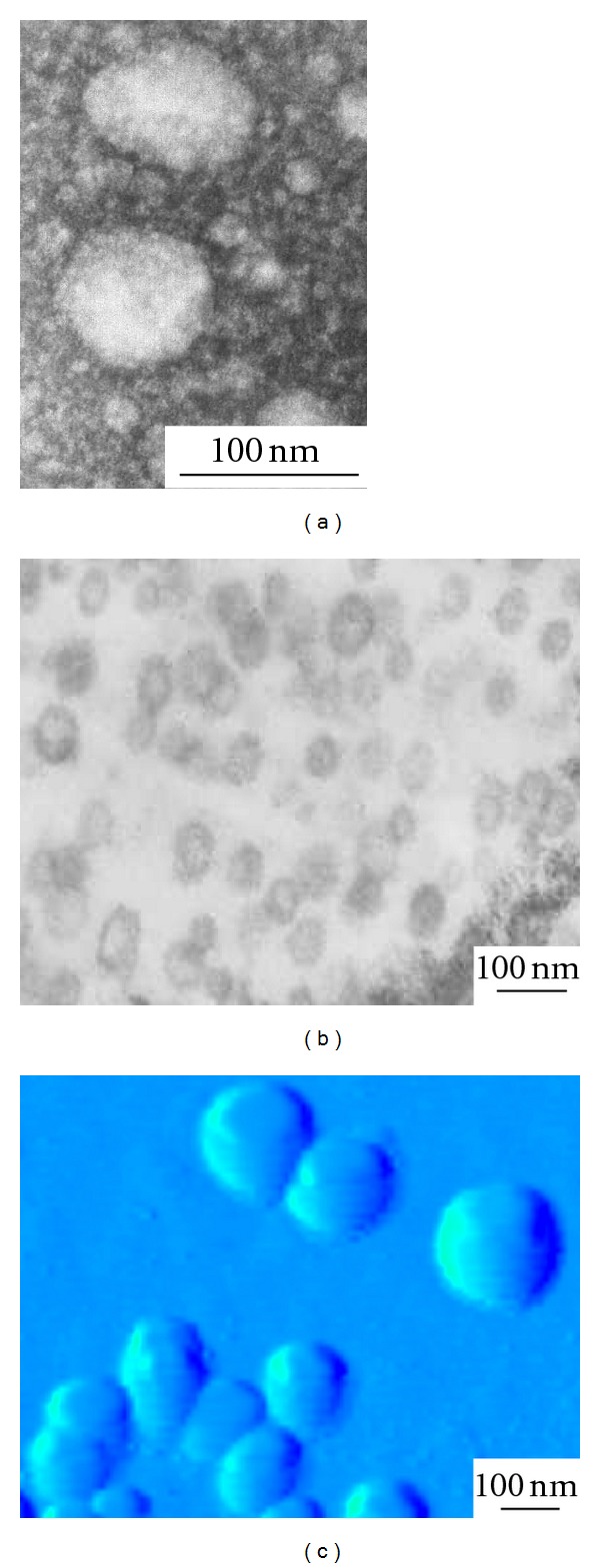
Images of EMVs of *A. laidlawii* PG8 obtained using transmission, (a, b) and atomic force (c) microscopy.

To test the ability of EMVs of *A. laidlawii *PG8 to penetrate from medium for *O. sativa* L. cultivation into tissues of overground parts of plants, special experiments were designed where rice plants grew in media with EMVs without mycoplasma cells (Figure 3), and PCR with the indicated primers was used to detect infects while TEM was used for analysis of plant ultrastructural organization.

**Figure 4:**
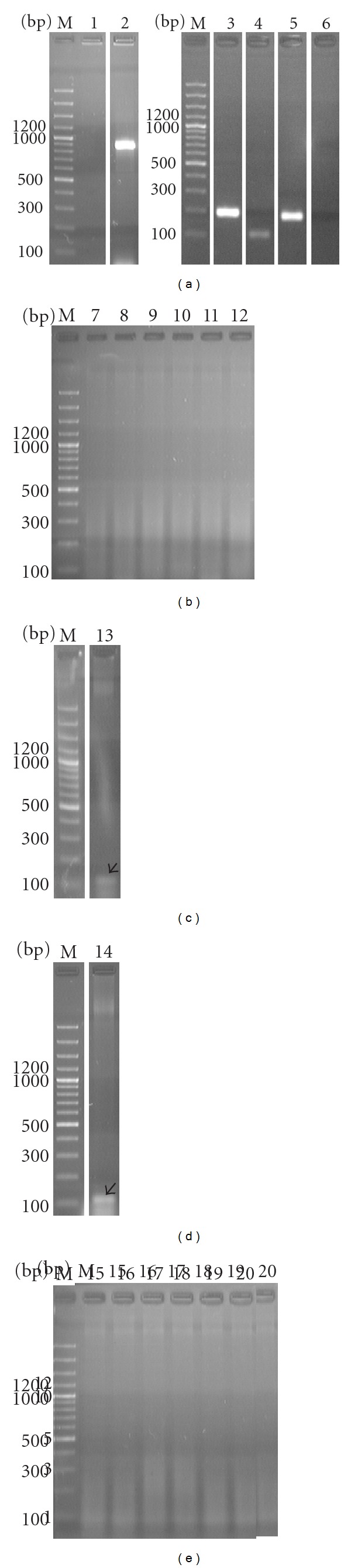
Electrophoregrams of amplification products for DNA nucleotide sequences of the following genes *fts*Z: (1, 7, 15), *pdh*C (2, 8, 16), *pnp *(3, 9, 17), *tuf*B (4, 10, 13, 14),* rpo*B (5, 11, 19), and 16S–23S rRNA (6, 12, 20) of *A. laidlawii *PG8 obtained at use, in PCR, total DNAs (as matrix) isolated from EMVs of *A. laidlawii *PG8 (a) and plant leaves grown in medium with EMVs (b and c—2 h and 9 days, resp.), the mycoplasma cells (d), and in the absence of the infect (e). Arrows indicate location of the corresponding PCR-signals. M—a marker of fragment lengths.

**Figure 5:**
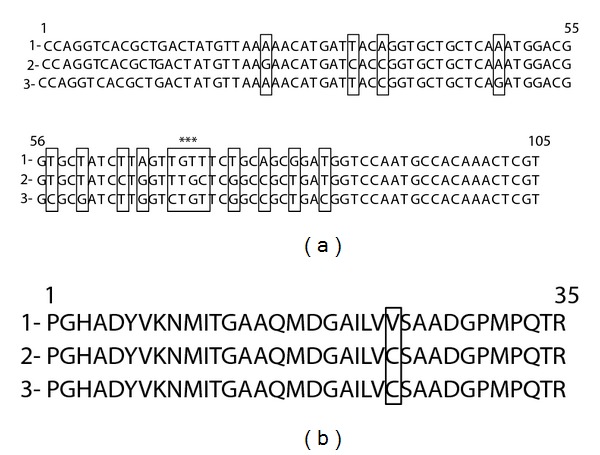
Results of alignment of the nucleotide (a) and amino acid (b) sequences of the fragment of *tufB*-gene of cells (1) and EMVs (2) of *A. laidlawii* PG8 as well as amplicon (3) obtained as a result of PCR with primers for detection of the nucleotide sequence of *tufB*-gene of *A. laidlawii* PG8 when DNA of plants grown in EMVs-containing medium was used as a matrix. Sequences of forward and reverse primers are indicated with italics. (□): nucleotide and amino acid replacements; *: changes at positions 70–72 results in replacing valine by cysteine.

Results of PCR with the use of matrix DNAs from *A. laidlawii *PG8 cells, EMVs, and plants, grown with and without vesicles, are presented at Figure 4. As it follows from the obtained data, 2 h since the beginning of plant incubation with EMVs, PCR signals for *pdh*C, *rpo*B, *pnp, tuf*B, *fts*Z,and 16S–23S rRNA were absent in the tested samples (Figure 4(b)). However, when primers for *tuf*B were used in plants grown for 9 days with EMVs as well as in plants cultivated with the mycoplasma cells, amplicon was registered that is similar in size to *tuf*B in EMVs of *A. laidlawii *PG8 (Figure 4(c)). Results of sequencing suggest on the belonging of this amplicon sequence to *tuf*B-gene of *A. laidlawii *(Figure 5). The detected nucleotide replacements are probably related to features of nucleotide sorting or/ and with subsequent DNA modifications in EMVs. However, this question should be clarified in the future. 

The obtained data may confirm the penetration of EMVs from medium to overground parts of plants. To detect the ability of mycoplasma EMVs to show virulent features linked with toxigenicity, we made analysis of plant response reactions toward EMVs persistence related with features of their ultrastructural organization. With this aim, transmission micrographies of ultrathin slices of *O. sativa *L. leaf tissues were investigated.

 It is known that toxigenicity of *A. laidlawii* is significantly related with the ability of the mycoplasma to induces a chronic oxidative stress [4]. At that, in plants that are not specific host for *A. laidlawii, *the mycoplasmamay induce inapparent infections when clear signs of morphologic anomalies (dwarfism, development of laterals, growth delay, icterus) are not evident but transcriptome and proteome reorganization, and alterations in tissue ultrastructure are present [2].

**Figure 6:**
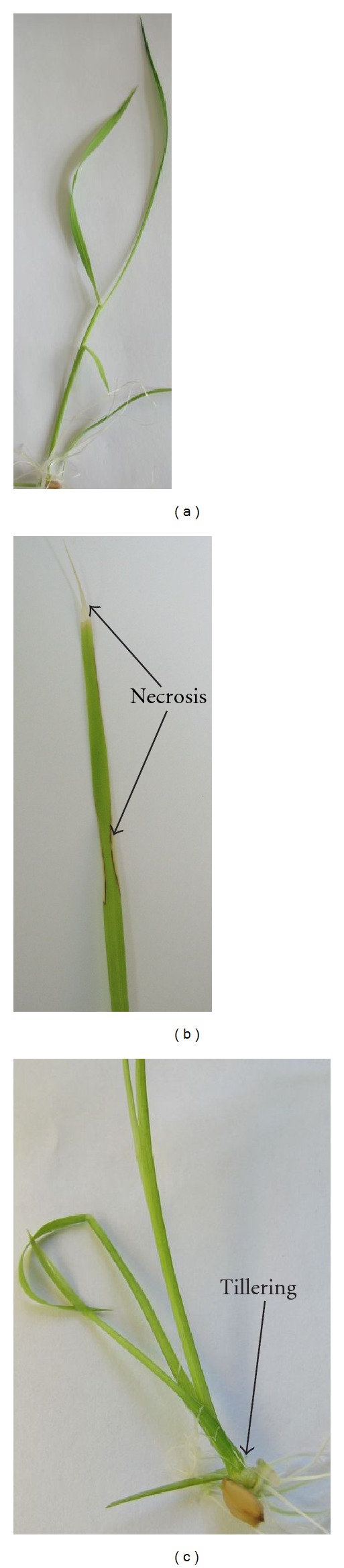
Morphologic alterations in plants (*O. sativa* L.) grown in medium with EMVs of *A. laidlawii *PG8.

**Figure 7:**
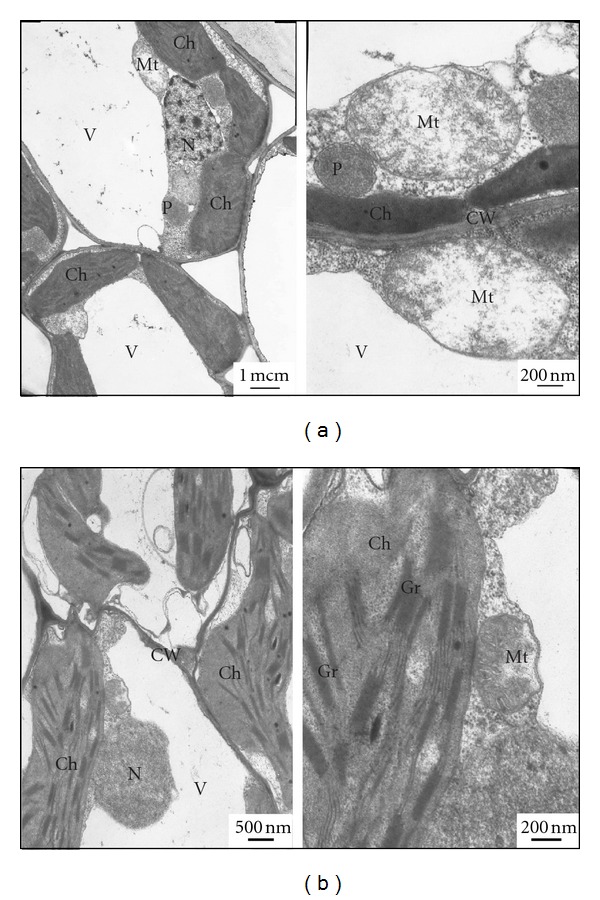
Ultrastructural organization of plant cells (*O. sativa* L.) grown in media with (a) and without (b) EMVs of *A. laidlawii *PG8. V: vacuole, Gr: grana, CW: cell wall, Mt: mitochondria, P: peroxisome, Ch: chloroplast, N: nucleus.

 As it was found in our studies, evident morphoses were not detected in plants grown in medium with EMVs of *A. laidlawii* PG8. Only in some plants, there were apical necrosis and tillering (Figure 6). However, we revealed significant alterations in tissue ultrastructure in plants grown in medium with EMVs (Figure 7): chloroplasts were located along the cell walls and did not contain amyloid grains; vacuoles of coat cells of vessels and parenchyma cells were fulfilled with soft content; mitochondria have brighten soft matrix with rare cristas. The observed alterations of ultrastructural organization of parenchyma cells in leaves of *O. sativa *L. are characteristic for plants infected with *A. laidlawii *PG8 cells [2] as well as under conditions of oxidative stress [15]. The obtained data may indicate that toxigenicity of EMVs as well the mycoplasma cells is primarily connected with the induction of oxidative stress. The detected similarity in response reactions of plants toward EMVs and the mycoplasma cells may be due to location of virulence factors at membranes of the infects [16]. The comparative analysis of the proteome profiling of membranes of cells and EMVs of the bacterium will probably be useful for justification of this statement.

Thus, as a result of our studies of interaction between *O. sativa* L. and EMVs secreted by *A. laidlawii *PG8 cells, it was presented for the first time that EMVs may display in plants the features of infect from the viewpoint of virulence criteria—invasivity, infectivity and toxigenicity—and favor bacterial phytopathogenicity. In this connection, detection of full content of *A. laidlawii* PG8 EMVs regarding proteins, lipids, and nucleic acids in different environments and careful analysis of pathogenicity of vesicles seem very actual from the viewpoint of fundamental biological research of the smallest prokaryotes as well as practical developments of controlling mycoplasma infections and contaminations of cell cultures and vaccines.
